# The multimodal nature of communicative efficiency in social interaction

**DOI:** 10.1038/s41598-022-22883-w

**Published:** 2022-11-09

**Authors:** Marlou Rasenberg, Wim Pouw, Asli Özyürek, Mark Dingemanse

**Affiliations:** 1 Centre for Language Studies, Radboud University, Nijmegen, The Netherlands; 2 Max Planck Institute for Psycholinguistics, Nijmegen, The Netherlands; 3 Donders Institute for Brain, Cognition and Behaviour, Radboud University, Nijmegen, The Netherlands; 4 Communicative Alignment in Brain and Behaviour Team, Language in Interaction Consortium, Nijmegen, The Netherlands

**Keywords:** Psychology, Human behaviour

## Abstract

How does communicative efficiency shape language use? We approach this question by studying it at the level of the dyad, and in terms of multimodal utterances. We investigate whether and how people minimize their joint speech and gesture efforts in face-to-face interactions, using linguistic and kinematic analyses. We zoom in on other-initiated repair—a conversational microcosm where people coordinate their utterances to solve problems with perceiving or understanding. We find that efforts in the spoken and gestural modalities are wielded in parallel across repair turns of different types, and that people repair conversational problems in the most cost-efficient way possible, minimizing the joint multimodal effort for the dyad as a whole. These results are in line with the principle of least collaborative effort in speech and with the reduction of joint costs in non-linguistic joint actions. The results extend our understanding of those coefficiency principles by revealing that they pertain to multimodal utterance design.

## Introduction

In joint actions, people coordinate their behaviors in order to achieve joint goals^1,2^. Whether people are moving a couch or having a chat, joint action appears to be organized according to a principle of efficiency or effort minimization^3–7^. Empirical work on joint action shows that this effort minimization appears to target overall *joint effort* (or *coefficiency*) rather than individual effort^8–10^. Work on spoken language likewise suggests that people work together to minimize the cost for the dyad as a social unit—known as *the principle of least collaborative effort*^11–13^. One consequence for the study of efficiency in language is that language use is not about idealized speakers producing optimal one-off utterances; instead, we need to consider the work that interacting participants jointly undertake to actively construe possible meanings.

The notion of coefficiency is in principle agnostic to the type of behavior involved. That is, joint action is recognized to involve a complex interplay of efforts exerted through various types and levels of behavior. However, when it comes to language use, efficiency is usually studied in *unimodal* ways (by focusing on written representations of speech), where communicative acts are considered to be *linear* (one word after the other). Complementary or parallel contributions across modalities are overlooked in accounts of efficiency in human languages^4,5^, despite the communicative capacities of composite utterances as revealed by research on multimodal interaction^14–18^. So, work on efficiency in coordinated spoken language use has yet to take into account how simultaneous articulators are concurrently employed to convey information (for work on sign language, see^19^). Here we take on the challenge to study how people efficiently coordinate multiple types of communicative behavior, by investigating if and how people minimize joint speech and gesture efforts in a task-based conversational setting.

We focus on stretches of conversation where people explicitly coordinate their utterances with the goal of jointly solving problems of perceiving or understanding—known as other-initiated repair^20,21^. In a typical sequence of other-initiated repair, one participant temporarily halts the conversation in order to ask for clarification with a repair initiation like “huh?” (open request), “who?” (restricted request), or “like this [gesture]?” (restricted offer)^22–26^, to which their conversational partner responds with a repair solution. After having jointly resolved the trouble, the participants end the repair sequence and the main conversation continues^27,28^. Repair initiations and solutions are defined strictly in terms of sequential positions in conversation, where the turns themselves can recruit any combination of communicative modalities^29–45^.

Sequences of other-initiated repair have played a key role in the development of the notion of least collaborative effort for English task-based and telephone interactions^12,13^ and in its generalization to co-present conversational interaction across diverse languages^46^. This work revealed that people collaboratively resolve trouble while minimizing their joint efforts in two ways. First, recipients who signal trouble prefer to use restricted formats (e.g., ‘Which one?’ or ‘You mean X?’) over open formats (e.g., ‘Huh?’), meaning that they initiate repair in the most specific way possible (the *specificity principle*). Second, the more specific the repair initiation, the longer the repair initiation (involving more speech effort), thereby minimizing the efforts needed for the sender to resolve the trouble in the repair solution (the *division of labor principle*). However, this prior work focused exclusively on unimodal utterances, either by classifying the referential formats of noun phrases, or by computing the orthographic length of turns. Gesture efforts in spoken language have been overlooked, even though prior research has shown that manual co-speech gestures can play an important role in repair initiations^38,42,47^ and repair solutions^31,32,48–50^. Since gestural efforts to convey meaning have not been incorporated in the division of labor equation, we cannot be sure that the speech-centered findings hold water for interactions in their true multimodal form.

Adopting a multimodal perspective is also warranted in light of recent studies showing that various interactional strategies (initially discovered based on speech-centered research) extend to the design of multimodal utterances. For example, people modulate both speech and gesture when trying to get a message across in noisy environments (multimodal Lombard effect^51^); adapt both speech and gestures to the degree of knowledge that is shared with a recipient (multimodal audience design^52^); and are likely to use cross-speaker repetition of both speech and gesture for establishing joint reference to novel objects (multimodal alignment^53^). These findings reinforce the notion that speech and co-speech gestures operate as part of an integrated system^54–56^, with people flexibly deploying and coordinating their use of both modalities to engage in joint meaning-making^42,53,57–62^.

In work on co-speech gesture, the notion of division of labor is sometimes used in reference to how effort is divided between the modalities of speech and gesture in one speaker’s utterances^63^. Here instead we focus on the dyad as a social unit, where our primary interest is how effort is distributed across contributions of different speakers, taking into account both speech and gesture.

To investigate the distributions of multimodal effort at the dyad level we focus on sequences of other-initiated repair in task-based interaction. Other-initiated repair has several properties that make it an ideal testing ground for studying efficiency in social interaction. First, it is a miniature coordination problem solved in real-time by two participants, making it a relevant domain for understanding joint actions more broadly^64^. Second, its sequential structure—an insert sequence composed of an initiation and proposed solution—is cross-linguistically well-attested and highly frequent^46,65^. Third, it comes in a small number of formats that we can compare in terms of multimodal effort and frequency of use. By tracking participants’ speech and gesture efforts in these conversational enclosures, we test whether *multimodal* contributions are optimized for least collaborative effort.

We use a director/matcher task in which people describe and find 3D shapes they have not seen before (Fig. 3). The shapes, displayed in a randomized array on two screens, are designed to present participants with coordination problems to be solved on the fly using multimodal communication. Standing face-to-face and instructed to communicate in any way they want, participants recruit multimodal utterances in relatively free-form interactions in order to negotiate mutual understanding and jointly solve the task. Speech and gesture behaviors were recorded with head-mounted microphones, cameras and markerless motion tracking devices.

For the spoken modality, we operationalized effort as the number of orthographic characters per repair turn, as this allows us to compare our findings to those of previous work^46^. Though not a direct measure of talk-in-interaction, orthographic length may be a reasonable proxy because (i) it is not affected by speech rate (while turn duration is) and (ii) it normalizes length across different speakers. In our dataset orthographic length strongly correlates with the duration of the repair turn (*r* = 0.93, *p* < 0.001), in line with earlier work^46^. For the gesture modality, we use the number of submovements of manual co-speech gestures. This has been used as a kinematic measure of complexity and effort before^51,66,67^ and measures akin to it have been shown to correlate with the number of information units in gestures as interpreted by human coders^66^. While perfect equivalence of measures across modalities is impossible, those proposed here are comparable in the sense that (a) both speech and co-speech gesture are used to negotiate meaning in other-initiated repair sequences, and (b) orthographic characters and submovements can both be used as a quantitative proxy for the amount of information that is (verbally or visually) conveyed by a repair turn.

We first investigate speech and gesture efforts separately, where we explore how these efforts are distributed across sequential positions (repair initiation and solution) and repair types (open request, restricted request and restricted offer). To investigate the division of multimodal effort between people, we compute a measure of multimodal effort by summing (standardized) speech and gesture efforts. We hypothesize that the type of repair initiation predicts how the joint amount of multimodal effort is divided between people, similarly to what has been found for the division of speech efforts^46^. That is, we hypothesize that the more specific the repair initiation (open request < restricted request < restricted offer), the higher the proportion of the multimodal cost paid in the repair initiation relative to the total cost paid in the initiation and solution together. Finally, in line with the principle of least collaborative effort^11–13^, we predict that people design their utterances so as to minimize the total amount of multimodal effort for the dyad as a whole. Specifically, we hypothesize that the repair type which yields the smallest amount of joint multimodal effort will be used most frequently.

## Results

Overall, 378 repair initiations were found in our dataset of task-based interactions from 20 dyads (comprising about 8 h of audio, video and motion tracking recordings in total). We found a mean of 18.9 repair initiations per dyad (*SD* = 9.92, *range* = 6–45), which amounts to a repair initiation occurring once every 1.5 min on average. There were 24 open requests, 39 restricted requests and 315 restricted offers.

### Speech and gesture effort

**Figure 1:**
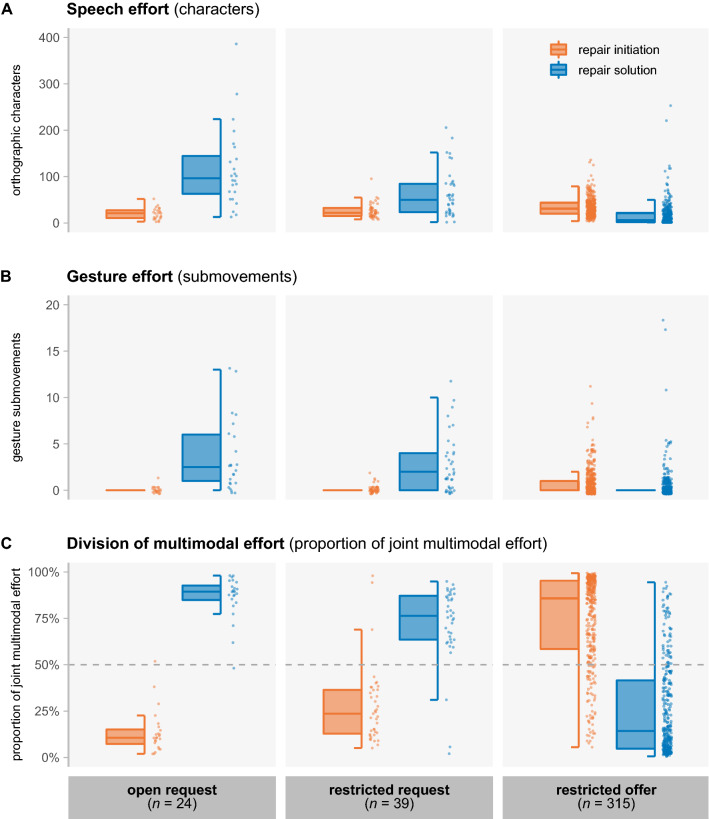
Boxplots showing the effort invested in the repair initiation (orange) and repair solution (blue), for repair formats of increasing specificity (open request < restricted request < restricted offer). The boxes represent the interquartile range; the middle line the median; the whiskers the minimum and maximum scores (outliers excluded). Every dot represents a repair initiation or solution. Absolute speech effort (**A**) and absolute gesture effort (**B**) both go up in repair initiations and down in repair solutions as repair formats become more specific. Proportional multimodal effort (**C**) shifts from repair initiation to repair solution as we move towards more specific repair formats. The dashed line represents equal division of effort across participants.

We start by reporting speech and gesture effort separately to allow for comparisons with prior unimodal work on the division of speech efforts in other-initiated repair^46^. The effort that people invest through the spoken modality to collaboratively resolve interactional trouble is shown in Fig. 1A. In repair initiations, speech efforts slightly increase as the type of initiation becomes more specific (open requests: *M* = 20.13, *SD* = 12.21; restricted requests: *M* = 27.05, *SD* = 16.63; restricted offers *M* = 34.38, *SD* = 20.18). In repair solutions, the opposite pattern emerges: when responding to more specific initiations, speech turns tend to become shorter (open requests: *M* = 116.63, *SD* = 87.18; restricted requests: *M* = 63.08, *SD* = 50.75; restricted offers *M* = 16.70, *SD* = 27.29). These findings are in line with the unimodal analyses in prior work^46^; see further notes on the division of verbal effort in Supplementary Information (S2).

For the gestural modality, there is considerable individual variation, with some people gesturing very rarely or not at all. In total, 479 co-speech gestures were produced across all repair initiations and solutions, with 37.2% of the turns containing at least one gesture. But the likelihood of encountering a gesture in a turn differs greatly across repair types and sequential positions; ranging from 4.2% in repair initiations of the type open request, to 79.2% in repair solutions in response to open requests. When quantifying gesture effort in terms of submovements, we find a similar pattern as for speech effort (Fig. 1B). As repair initiations become more specific, more gesture submovements are used in the initiation (open requests: *M* = 0.04, *SD* = 0.20; restricted requests: *M* = 0.15, *SD* = 0.43; restricted offers *M* = 1.02, *SD* = 1.57), and fewer are used in the solution (open requests: *M* = 3.63, *SD* = 3.84; restricted requests: *M* = 2.67, *SD* = 3.15; restricted offers *M* = 0.53, *SD* = 1.74).

### Division of multimodal effort

In accordance with the inherently multimodal nature of our dataset, we analyze how the total amount of multimodal effort is divided across participants (Fig. 1C). Multimodal effort is the sum of the effort invested through the two available modalities, so it can be speech-only or speech-plus-gesture (we found no cases of gesture-only). Adopting a narrow notion of multimodality (focusing on visual information in manual co-speech gestures only), we can consider 63% of the repair sequences as being multimodal in nature, containing one or more gestures by at least one of the participants.

We find that the proportional cost paid by the person initiating repair versus the person resolving the trouble varies as a function of the repair type, and deviates from a case of equal division (where each person would invest 50% of the total effort). The proportion of multimodal effort invested in the repair initiation was higher for restricted requests compared to open requests (β = 0.14, *SE* = 0.06, *t* = 2.35, *p* = 0.02), and higher for restricted offers compared to restricted requests (β = 0.47, *SE* = 0.04, *t* = 11.42, *p* < 0.001), as revealed by mixed effects models (with random intercepts for dyads). Overall, we find a trade-off between the efforts invested by the two members of the dyad; the more multimodal effort is invested by the person initiating repair, the less multimodal effort is used to respond to it (*r* = − 0.14, *p* = 0.007).

### Minimization of joint multimodal effort

**Figure 2:**
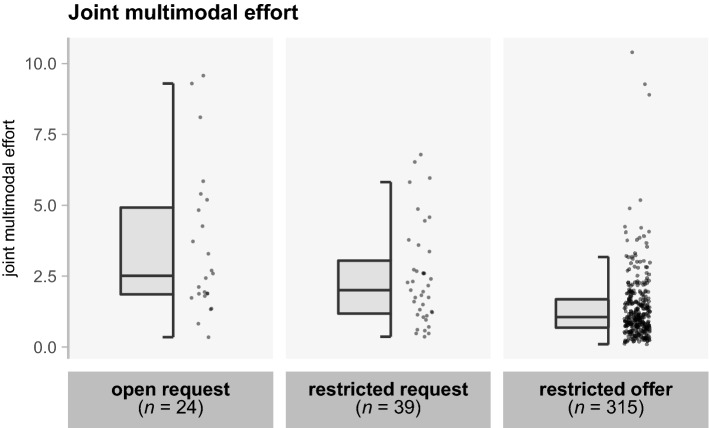
Boxplots showing the joint amount of multimodal effort invested by both participants to resolve the interactional trouble. The boxes represent the interquartile range; the middle line the median; the whiskers the minimum and maximum scores (outliers excluded). Every dot represents a repair sequence, i.e., repair initiation and repair solution together. As the specificity of repair formats goes up, joint multimodal effort invested goes down.

The total amount of multimodal effort that was invested by the dyad to resolve interactional trouble (in the repair initiation and repair solution combined) is shown for each repair type in Fig. 2. On average, we find that the joint multimodal effort is smallest when the repair initiation was a restricted offer. A mixed effect model (with random intercepts and slopes for dyads, and random intercepts for target items) revealed that joint effort is less in sequences involving restricted offers (*M* = 1.37, *SD* = 1.21) compared to restricted requests (*M* = 2.45, *SD* = 1.74; β = -1.15, *SE* = 0.30, *t* = -3.80, *p* = 0.006), but that joint effort in restricted requests does not differ significantly from open requests (*M* = 3.51, *SD* = 2.58; β = -0.86, *SE* = 0.55, *t* = -1.58, *p* = 0.137). In terms of how often the different types of repair initiations are used, we found that restricted offers are by far the most frequent (83,3%). A mixed effect model (with random intercepts for dyads and target items) revealed that restricted offers occur significantly more than restricted requests (10,3%; β = 0.91, *SE* = 0.06, *t* = 15.89, *p* < 0.001), while restricted requests do not differ in frequency from open requests (6,3%; β = 0.05, *SE* = 0.06, *t* = 0.82, *p* = 0.413). The preference for using restricted offers paired with the finding that these repair types yield the lowest amounts of joint effort thus means that people appear to do repair in the most cost-efficient way possible, minimizing the joint multimodal effort for the dyad as a whole.

## Discussion

The present study investigated how language use is shaped by communicative efficiency from a multimodal and interactional perspective. We focused on short time windows in turn-by-turn interaction where people work together to achieve a particular joint goal: repairing a problem with perceiving or understanding talk. We analyzed how speech and co-speech gesture efforts are distributed across repair types (open requests, restricted requests and restricted offers) and sequential positions (the repair initiation and the repair solution), with the aim to test whether the division of multimodal effort is optimized for least collaborative effort.

There are three main findings. First, we find that speech and gesture efforts rise and fall together across repair types and sequential positions. This corroborates the view that speech and gesture are integral parts of a single multimodal communicative system^54–56,68^, and matches speech-gesture parallelism as reported for other interactional phenomena; for example, people increase both speech and gesture efforts in noisy environments^51^, and people use fewer words and fewer gestures as common ground increases (for a review, see^52^).

Second, we show in detail how people orchestrate efforts in speech and gesture to achieve rapid coordination. In particular, the type of repair initiation used predicts how people divide their multimodal efforts: the more specific the repair initiation, the more multimodal effort is invested by the person initiating repair, leaving less work for the sender of the original message to resolve the trouble. This replicates prior unimodal work showing systematicity in how verbal effort is distributed across repair initiation and solution^46^, and shows that the pattern is robust enough to hold in both naturalistic as well as task-based conversations. Our results extend this division of labor principle to composite utterances, providing an unprecedented view of multimodal contributions to the coordination of joint action.

Third, we find that people overwhelmingly converge on the repair format (i.e., restricted offer) that minimizes multimodal effort for the dyad as a social unit. This is a novel, direct attestation of the principle of least collaborative effort^11–13^ that is made possible by combining quantifications of speech effort with new, reproducible methods to measure gestural effort in terms of kinematics.

Taken together, these findings provide a novel unifying perspective on studies of language use^11–13^ and non-linguistic joint action^8–10^. The coordination of joint action minimally involves dynamically updated task representations, monitoring processes, and adjustable behaviors^69^. Although linguistic coordination has sometimes been cast as a qualitatively different form of coordination^69^, here we have shown that the micro-environment of interactive repair—which occurs at frequencies and timescales more commensurate with joint action—provides a unique window onto the real-time negotiation of distributed agency. In repair, people provide public evidence of representations and monitoring processes, allowing them to rapidly hone in on optimal coordinative solutions^70^. By zooming in on these miniature coordination problems, we reveal systematicity in how people adjust multiple types of communicative behavior to minimize joint efforts, thereby unravelling the multimodal nature of coefficiency in conversational joint action.

Beyond the empirical findings, our study also makes conceptual and methodological contributions. One is to extend and enrich standard notions of efficiency in language use. Prior work has usually considered efficiency in terms of unimodal message length^4^. One limitation of such operationalizations is that they easily lose sight of the fact that in conversation, the interactional work of achieving mutual understanding is often distributed across turns and participants^71,72^. We argue that communicative effort and efficiency are best studied at the level of the dyad as a social unit, and we show how interactive repair provides a microcosm that allows us to study the public negotiation of mutual understanding over multiple turns.

Another challenge of the most common unimodal operationalizations is that, when applied to co-present conversational settings, they are incomplete and reductive, focusing on language as a unimodal discrete symbol system while overlooking multimodal, continuous and dynamic properties of language use^66,73^. Our contribution towards solving this challenge consists of using methods and insights from studies of joint action and behavioral dynamics^74–76^. Our measures capture how people use both categorical and gradient semiotic resources in multiple modalities to make meaning together, where we operationalized effort with (a) a linguistically informed quantification of speech in terms of orthographic characters^46,77^ and (b) a kinematic measure of submovements derived from continuous manual movement^66,67,78,79^. These measures do not fully capture the multi-semiotic dimension of social interaction, as we disregard dynamic properties in the spoken modality (see e.g., research on the phonetic characteristics of repair solutions^80^) as well as non-manual embodied resources (further discussed below). However, we believe the combination of measures for the spoken and gestural modality used in the present study are a step in the direction of a truly multimodal linguistics that takes semiotic diversity seriously^54^.

### Limitations

The nature of our task may have invited more representational gestures than some other conversational settings, as the 3D objects lack conventional names and lend themselves well to iconic depiction^81^. We also found relatively high amounts of restricted offers (83,3%), compared to restricted requests (10,3%) and open requests (6,3%; a pattern reported for other task-based datasets as well^82,83^). This might partially follow from the lab setting, in which people were unlikely to be troubled by noise interference or other low-level perceptual problems (which are associated with open requests^46^). Perhaps because of the overall low amounts of open and restricted requests, we found no statistical difference in the frequency of these two types. Consequently, our conclusion that people repair trouble in the most cost-efficient way possible is based on the use of restricted offers: repair sequences of this type are the most frequent *and* require the smallest amount of joint multimodal effort. Future research could test whether frequency and cost-efficiency patterns together across all repair types in more naturalistic settings. Though the lab- and task-based setting can affect the use of gesture and repair types, our findings are largely in line with studies on other-initiated repair in more naturalistic settings, where people also prefer to use restricted formats over open requests^46^, and take advantage of iconic properties of gestures for repair initiations and solutions^42,47,49^. We therefore believe that the observed distribution of multimodal effort across repair types is likely to be robust enough to generalize to everyday language use. That is, while we might expect to find fewer manual gestures and more open and restricted requests, we would still predict people’s multimodal productions to be more effortful in repair initiations of the type restricted offer compared to open and restricted requests (and vice versa for solutions).

We have investigated the cost-efficient use of words and manual co-speech gestures. By foregrounding efficiency in a task-based setting and focusing on speech and gestures, we have of course captured only a partial view of what it means for people to coordinate their multimodal utterances to resolve conversational problems. Future studies could broaden this view, for example by incorporating eye gaze, eyebrow movements, head movements and forward leans, which are known to play a role in signaling trouble^29,30,33,36,40,41,84^. This could be complemented by a consideration of other social or expressive factors which influence communicative behaviors, as people can for example opt to use an open request rather than a restricted offer for face-saving purposes^85^. More empirical and theoretical work is needed to understand how pressures and constraints in human sociality interact with principles of efficiency in joint action. We take this to be an important avenue for future research, where moving our attention from efforts of the individual to those of the dyad as a cooperative social unit is an important first step.

### Conclusion

In summary, in this study we investigated communicative efficiency from a multimodal and interactional perspective by zooming in on other-initiated repair sequences. As a conversational environment in which there is a clear goal, a limited set of turns to reach that goal and a limited inventory of communicative resources to use in those turns, other-initiated repair is a natural laboratory for the systematic study of coefficiency in language use. Our findings reveal that people divide the total amount of multimodal effort between them in such a way as to minimize the overall amount of speech and gesture efforts for the dyad as a whole. By investigating how communicative efficiency is realized in multimodal language use at the level of the dyad as social unit, we have shown how minimizing effort in language use is an interactional achievement.

## Methods

### Participants

Twenty dyads took part in the study (10 mixed-gender, 6 female-only and 4 male-only dyads, *M*_age_ = 22.3 years, *Range*_age_ = 18–32 years). The unacquainted participants were recruited via the Radboud SONA participant pool system. Participants provided informed consent and were paid for participation. The participants who are visible in the figures provided informed consent to publish the images in an online open access publication. The study met the criteria of the blanket ethical approval for standard studies of the Commission for Human Research Arnhem-Nijmegen (DCCN CMO 2014/288), and was conducted in accordance with relevant guidelines and regulations.

### Apparatus and materials

**Figure 3:**
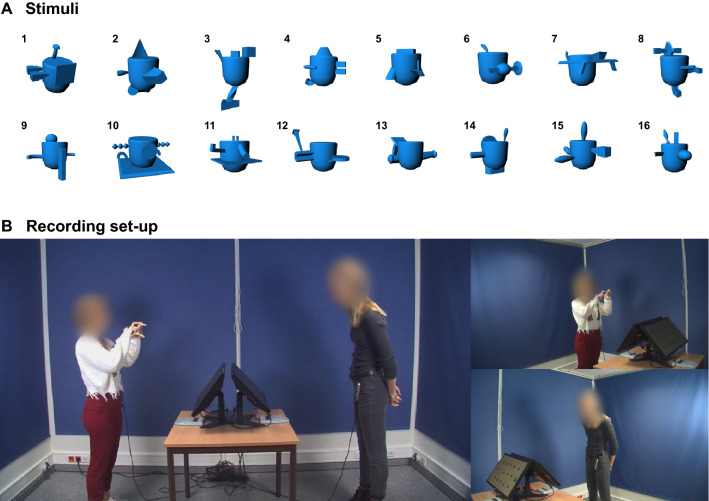
Panel (**A**) shows the “Fribbles” that were used as stimuli. Panel (**B**) shows the set-up by means of screenshots from the three cameras.

The stimuli were 16 images of novel 3D objects, called ‘Fribbles’ (adapted from Barry et al.^86^), see Fig. 3A. During the interaction, participants were standing face-to-face, where each had their own button box and screen (24′ BenQ XL2430T), slightly tilted, and positioned at hip height to ensure mutual visibility of upper torso and gesturing area (see Fig. 3B). The Fribbles were presented on these screens on a grey background in rows of 5, 6, and 5 figures respectively, in a size of about 4 × 4 cm per figure, with a corresponding label next to it. The order of the Fribbles was random and varied for the participants (but was constant across dyads). Audio was recorded with head-mounted microphones (Samson QV) and videos were made with three HD cameras (JVC GY-HM100/150). 3D motion tracking data was collected using two Microsoft Kinects V2 (for 25 joints, sampled at 30 Hz).

### Procedure

Participants were assigned director/matcher roles. In each trial, a red triangle highlighted a single target Fribble on the director’s screen. The participants were instructed to communicate in order for the matcher to find the target item on their screen. To indicate their selection, the matcher said the corresponding label out loud and pressed a button to go to the next trial, where the participants switched director/matcher roles. After matching all 16 Fribbles, a new round would start; in total six rounds were completed, yielding a total of 96 trials. No time constraints were posed and the participants did not receive feedback about accuracy. Participants were told that they were ‘free to communicate in any way they want’ (an instruction phrased to be agnostic about communicative modality, i.e., speech and/or gesture), and that their performance would be a joint achievement. Dyads spent 24.4 min on the task on average (*range* = 14.2–34.6 min).

### Analysis

The audio–video data were annotated in ELAN (version 5.8); data processing and statistical analyses were performed with the R statistical program (version 4.0.2).

Speech was segmented into Turn Constructional Units (TCUs; i.e., potentially complete, meaningful utterances^87–89^) and orthographically transcribed based on the standard spelling conventions of Dutch. Other-initiated repair was coded based on a modified version of the coding scheme by Dingemanse et al.^90^. We annotated the trouble source, repair initiation and repair solution, where the boundaries of those annotations corresponded to the speech annotations (where a single repair annotation could correspond to a single TCU or span multiple TCUs). Subsequently, repair initiations were categorized into three types: *open request, restricted request* and *restricted offer*. Details on the coding procedure including examples can be found as Supplementary Information (S1.1). Inter-rater reliability for repair identification, segmentation and coding was moderate to high (all yielded minimally 75% agreement; for details and additional reliability measures, see Supplementary Information; S1.2).

For co-speech gestures, the stroke phase was annotated for gesture units (i.e., the meaningful part of the gestural movement^16,56^), for the left and right hand separately. Inter-rater reliability was substantial for gesture identification, segmentation and coding (minimally 75% agreement; for details and additional measures, see Supplementary Information; S1.3). We considered gestures to be part of a repair turn when the gesture stroke completely overlapped with the repair annotation (which was the case for 91.5% of the gestures), but used fine-grained rules and manual inspection in case of partial or no overlap (see Supplementary Information; S1.4). All types of manual co-speech gestures were included in the analysis, but the majority of the gestures in the dataset are iconic (89.8%).

**Figure 4:**
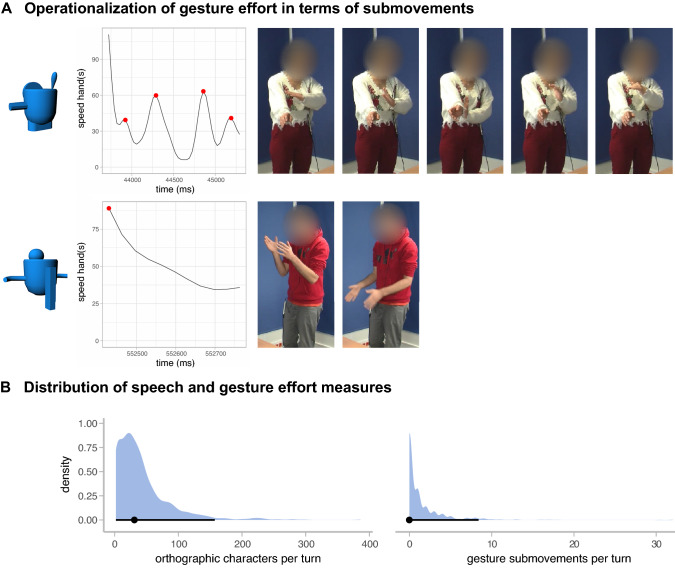
In panel (**A**), the top row shows a gesture which depicts the subpart on the left side of the Fribble. The gesture is produced by a matcher as part of a restricted offer with the following speech: “ah en is zijn arm uh rond maar ook een beetje met hoeken?” [literal translation: *ah and is his arm round but also a bit with corners?*]. The right arm is extended to model the ‘arm’, while the left hand is moved around it to depict the angular shape (number of submovements: 4). The bottom row shows a gesture which was produced by a director in a repair solution in response to a restricted offer. The gesture depicts the rectangular subpart on the front side of the Fribble, while saying “ja precies” [*yes exactly*]. The multimodal utterance thereby confirms the preceding restricted offer (which contained an identical gesture). The hands are kept somewhat apart (to depict the width of the rectangle), and moved straight downwards (number of submovements: 1). The density plots in panel (**B**) show the distributions for the speech and gesture effort measures (the dots are the median and the lines the 95% quantile interval).

Submovements were computed for each gesture stroke, of which the onset and offsets were determined by the manual annotations. The calculation was based on the position of the left- and right-hand tips in 3D space. To ignore noise-related jitter, we smoothed the position traces, and their derivatives (3D speed) with a third order Kolmogorov–Zurbenko (KZ) filter with a span of 2. 3D gesture speed was used to determine submovements, which was based on the speed of the left or right hand for one-handed gestures, or the summed speed of both hands in the case of two-handed gestures. The number of submovements was then computed by identifying local maxima peaks in the 3D speed time series^66,79^. To this end, we used R-package *pracma* and considered a peak to be a submovement when it exceeded at least 10 cm/s and only if they had at least 100 ms distance between adjacent peaks. The minimum amount of submovements per gesture stroke is 1 (i.e., static strokes are considered to consist of 1 submovement). Two examples of gestures along with their submovement profile are presented in Fig. 4A (for more examples, see Supplementary Information; S1.5).

In order to analyze the division of multimodal effort, we combined the speech and gesture efforts to yield a multimodal effort variable. We first standardized the individual speech and gesture measures (as their distributions differed greatly, with gesture submovements being zero-inflated, see Fig. 4B), and then summed them. We then calculated the proportion of multimodal effort in the repair initiation as compared to the total multimodal effort in the repair initiation and repair solution. To subsequently inspect how the total amount of joint effort varies across repair types, we summed the multimodal effort in the repair initiation and repair solution for each repair sequence. The resulting measures (i.e., the proportion of effort in the repair initiation and total joint effort) were used as dependent variables in mixed effects models with random intercepts and slopes for dyads and target items (unless reported otherwise, when a maximal model was not possible due to convergence issues) and repair initiator type as predictor. We used backward difference contrast coding to compare restricted requests to open requests, and restricted offers to restricted requests.

## Supplementary Information

Supplementary Information.

## Data Availability

All data and analysis scripts used for this study are openly available on the Donders Repository at: 10.34973/12dp-9q56.
