# Supplementary material for: The multimodal nature of communicative efficiency in social interaction
Source: Sci Rep. 2022 Nov 9;12:19111. doi: 10.1038/s41598-022-22883-w (PMC9646718; doi:10.1038/s41598-022-22883-w)
Supplement: Supplementary file 1 — Supplementary Information. [file 41598_2022_22883_MOESM1_ESM.pdf]

Supplementary Information

For the paper:

The multimodal nature of communicative efficiency in social interaction

Marlou Rasenberg* [a,b,d] [0000-0003-1812-6907], Wim Pouw [b,c,d] [0000-0003-2729-6502], Asli Özyürek [a,b,c,d] [0000-0003-4914-2643] and Mark Dingemanse [a,d] [0000-0002-3290-5723]

[a] Centre for Language Studies, Radboud University
[b] Max Planck Institute for Psycholinguistics
[c] Donders Institute for Brain, Cognition and Behaviour, Radboud University
[d] Communicative Alignment in Brain and Behaviour team, Language in Interaction Consortium, the Netherlands

*corresponding author: Marlou Rasenberg, Max Planck Institute for Psycholinguistics, P.O. Box 310, 6500 AH Nijmegen, the Netherlands, marlou.rasenberg@mpi.nl

Table of contents

References

S1 Method details

S1.1 Repair coding

S1.2 Inter-rater reliability for repair coding

S1.3 Inter-rater reliability for gesture **coding**

S1.4 Linking gestures to repair annotations

S1.5 Operationalization of gesture effort: more examples

S2 Results details

References

S1 Method details

S1.1 Repair coding

We code *other-initiated self-repair*, which are practices that interrupt the ongoing course of action to attend to possible trouble in speaking, hearing or understanding the talk[1,2]. The coding for this study is based on the coding scheme by Dingemanse, Kendrick and Enfield[3].

We distinguish the following elements (henceforth 'repair turns'):

- TROUBLE SOURCE (T-1)
- REPAIR INITIATION (T0)
- REPAIR SOLUTION (T+1)

For a turn to be considered a T0, it needs to be preceded by a T-1 and followed by a T+1. T0s are coded as being one of three (mutually-exclusive) formats:

- OPEN REQUEST. An expression that requests clarification of a prior turn, leaving open where or what the problem is. Often an interjection or 'What?'-like form; typically results in repetition.
- RESTRICTED REQUEST. An expression that requests specification or clarification, restricted to a specific element of the trouble source. Often includes WH-question word and/or repetition.
- RESTRICTED REQUEST. A polar question that offers a candidate understanding and invites confirmation or correction in the next turn. Can include repetition and/or new material.

In order to identify and code other-initiated repair, we rely on the whole multimodal interactional context. That is, besides speech productions (what people say and how they say it), we also rely on participants' co-speech gestures, visual-bodily behavior (e.g., eye gaze) and the stimulus items during coding.

A T0 may occur on its own or as part of a more extended (non-minimal) repair sequence. For minimal sequences, we coded T-1, T0 and T+1. For non-minimal sequences, we coded only the first/original T-1 and all T0s and T+1s. All T0s and T+1s were incorporated in the analyses.

We created annotations for the repair turns in ELAN (version 5.8), in such a way that they temporally corresponded to the speech annotations. Remember that speech was segmented on the level of Turn Constructional Units (TCUs; i.e., potentially complete, meaningful utterances[4–6]). A repair turn could consequently correspond to a single TCU or span multiple TCUs (see examples below). The repair annotations were created in such a way that the boundaries of each repair turn correspond to the onset and offsets of the speech annotations. For information on how gestures were linked to these repair annotations, see S1.4.

Below we present some examples. Underlined text is the speech which temporally overlapped with the gesture stroke; square brackets indicate overlapping speech onsets.

Example 1: OPEN REQUEST		
T-1	A (director):	um is een kopje met een n- neus aan de rechterkant en een vierkante piercing er eigenlijk<br>*um is a cup with a n- nose on the right side and a square piercing there actually*
T-1	A:	halve wijnglas<br>*half wineglass*
T-1	A:	en een kleine antenne bovenop<br>*and a small antenna on top*
	B	uh uh<br>uh uh
T0	B:	#laughs# wat?<br>*what?*
T+1	A:	dus gewoon één balletje zit aan de voorkant [gesture]<br>*so just one ball is on the front* [gesture]
T+1	B:	j[a<br>*y[es*
T+1	A:	[en die heeft zo'n [gesture] eigenlijk wel zo'n [gesture] piercing erdoorheen<br>*[and that has such a [gesture] actually such a [gesture] piercing through it*

Example 2: RESTRICTED REQUEST		
T-1	A (director):	dit is de hoofdvorm waarbij um er rechts [gesture] uh een cirkeltje is gewoon zo plat [gesture]<br>*this is the main shape where um on the right [gesture] uh a circle is just flat 'like this' [gesture]*
	A:	links heb je die vorm die half uitgesneden is met een soort spitse punt erin<br>*on the left you have that shape that is half cut out with a sort of pointed point in it*
T0	B	rechts is een ?<br>*on the right is a ?*
T+1	B:	ja een cirkeltje [gesture] die eraan vast is geplakt waar je *iets aa- op* [gesture] kan zetten<br>*yes a circle [gesture] that is pasted on it where you can put something a- on [gesture]*

Example 3: RESTRICTED OFFER		
T-1	A (director):	die met die hoek zegmaar [gesture] aan de onderkant<br>*the one with that hook so to say [gesture] on the bottom*
T0	B:	ja die zo [gesture] [uh<br>yes that 'like this' [gesture] [uh
T+1	A:	[ja ja ja<br>*[yes yes yes*

S1.2 Inter-rater reliability for repair coding

To establish inter-rater reliability for repair coding, two coders independently coded 20% of the complete dataset (384 trials, *n*=74 repair comparisons).

Identification of repair

When inspecting agreement on other-initiated repair identification on the trial level, we found that coders identified the same amount of repair initiations in 94.3% of the trials. The inter-rater reliability for the number of identified repair initiations per trial (ICC = 0.88) was deemed adequate[7]. When inspecting agreement on a case-by-case level, we found that in 45 cases the coders agreed on the identification of a repair initiation (i.e., both coders independently coded a turn as an initiation), but disagreed in 29 cases (i.e., one coder considered a turn an initiation while the other did not). So, initial inter-rater agreement on the identification of repair initiations was 60.8%. We examined the underlying pattern of divergence and found that many disagreements were related to a specific category of the coding scheme. These are cases where coding comments indicated that it was hard to judge whether a prior turn of the partner is treated as problematic or whether the participant is instead requesting additional information or demonstrating understanding. For example:

TROUBLE SOURCE	A: uh bovenop het kopje heb je een soort driehoek of eigenlijk is het een vierkantje maar je ziet niet helemaal meer het puntje ervan en daar doorheen zit er iets doorheen gestoken
	A: uh on top of the cup you have a sort of triangle or actually it is a square but you cannot fully see the top of it anymore and then through that there is something put through it
REPAIR INITIATION	B: ja dus op de bovenkant zit zo'n ruitvormige
	B: yes so on top there is a diamond shaped
REPAIR SOLUTION	A: ja
	A: yes

The coders jointly identified these cases in the reliability coding set (relying on the coding comments in ELAN). When excluding these 14 cases from the reliability set, the agreement on the identification of other-initiated repair was 75%. Subsequently, to obtain reliable and systematic coding for cases belonging to this category, we decided to opt for an inclusive approach; i.e., include all of these cases as other-initiated repair whilst (re)coding the data.

For the previous reports on agreement on identification, we scored how many repair initiation annotations overlapped, where we disregarded differences in the length of the annotations and/or the number of segments for trouble source, repair initiation and repair solution. For example, one repair initiation annotation from one coder could span two repair initiation annotations of the other coder, or a trouble source annotation from one coder could consist of two TCUs while the other coder's annotation consisted of only one TCU. For the subset of 45 repair initiations that were identified by both coders, the degree of organization of the coder's segmentations of the repair sequences were assessed with the Staccato algorithm[8,9]. This resulted in scores of 0.90, 0.96 and 0.94 (on a scale from -1 to 1) for troubles, repair initiations and repair solutions respectively – indicating that the

coders had highly similar understandings of how the observed repair sequences had to be segmented. In terms of percentages:

- for trouble sources there was 75,9% complete, 24,1% partial and 6,9% no overlap in the annotations;
- for repair initiations there was 95,6% complete and 4,4% partial overlap in the annotations (no overlap is NA);
- for repair solutions there was 84,4% complete, 13,3% partial and 2,2% no overlap in the annotations.

Repair type

Inter-rater agreement for repair initiator type (agreement = 84.4%, Cohen's kappa = .58) was deemed adequate. Note that this variable has a skewed distribution, yielding a lower Kappa value despite a relatively high percentage agreement score – known as the 'high agreement, low consistency' paradox[10,11]. The main discrepancy in coding repair type could be attributed to a difficulty in differentiating two types of restricted formats, as shown by the fact that agreement on *restricted* versus *open* formats was almost perfect (agreement = 97.8%, Cohen's kappa = .88). The divergence within the restricted formats largely resulted from a different understanding of a small set of repair initiations in which the trouble source was (partially) repeated, which affected whether these cases were allotted to *restricted offer* (the largest category to begin with) or *restricted request* (relatively rare). This was resolved through discussion; the coders arrived at a common understanding of these cases as 'trouble-presenting repeats'[12], which were subsequently (re)coded as restricted offers.

S1.3 Inter-rater reliability for gesture coding

To establish inter-rater reliability for gesture coding, we focused on the first two rounds of the interaction (where presumably the most (diverse) gestures would occur). Two coders independently coded 96 trials (i.e., 5% of the 1920 trials in the total dataset; and 15% of the trials in round 1 and 2), yielding a comparison of n = 296 gesture annotations. Inter-rater agreement on gesture identification was 89.2%. For this measure, we scored how many annotations overlapped, where we disregarded differences in handedness, the length of the annotations and/or the number of segments (e.g., one stroke annotation from one coder spanning two stroke annotations of the other coder). To also assess these aspects of the degree of organization of the coder's segmentations, we used the Staccato algorithm [8,9]. We applied this to the left and right hand of each participant separately, which resulted in scores of 0.77, 0.71, 0.80 and 0.75 (on a scale from -1 to 1) – indicating that the coders had similar understandings of how the observed gestures had to be segmented. Inter-rater agreement for gesture type was substantial (agreement = 95.1%, Cohen's kappa = .64).

S1.4 Linking gestures to repair annotations

Since we annotated gestures and repair annotations separately, we still needed to 'link' these, in order to quantify the use of gestures in repair sequences. Note again that the repair annotations corresponded to the speech annotations (which were segmented into TCUs), and thus we will need to identify which gestures correspond to the spoken repair annotations (i.e., trouble source, repair initiation and repair solution). Though in principle it is possible for people to initiate or resolve repair through gesture alone (i.e., without any speech production), we did not encounter this in our dataset.

Co-speech gestures tend to have a tight temporal link to speech, meaning that gestures are usually produced simultaneously with or slightly before the production of the co-expressive speech[13–15]. For our dataset this entails that when people gesture while initiating or resolving repair, that these gestures are likely to overlap in time with the spoken repair utterances. However, it is also possible for a gesture to only partially overlap with the co-expressive spoken utterance, or to even completely precede or follow a spoken utterance, i.e., to be produced in silence[16,17]. Consequently, gestures that correspond to a particular repair annotation might be produced shortly before or after the spoken turn, and should thus also be included in the present study (i.e., linked to the corresponding repair annotation).

Figure S1.4 visualizes which gestures we have included in the dataset. As the general rule, we considered gestures to be part of a repair turn when the gesture stroke completely overlapped with the repair annotation (see row 1 in Figure S1.4). In case of partial overlap, we included the gestures when the stroke overlapped more than 50% (rows 2 and 3). If the overlap was smaller (<50%), but the stroke *did not* overlap with any prior or next speech, we also included the gesture (rows 4 and 5). In case of small overlap (<50%) where the stroke *did* overlap with a prior or next speech turn, we manually inspected those cases to see which turn was most co-expressive with the gesture, and decided whether or not to link those gestures to the repair annotation (rows 8 and 9). Finally, there might be no overlap at all between the gesture stroke and a repair annotation. For those gestures, if they preceded or followed the repair turn by maximally 2000 milliseconds, and if they *did not* overlap with any prior or next speech, we again manually inspected whether they corresponded to the repair annotation and (de)selected them for inclusion (rows 6 and 7). If they *did* overlap with other speech turns, we excluded them.

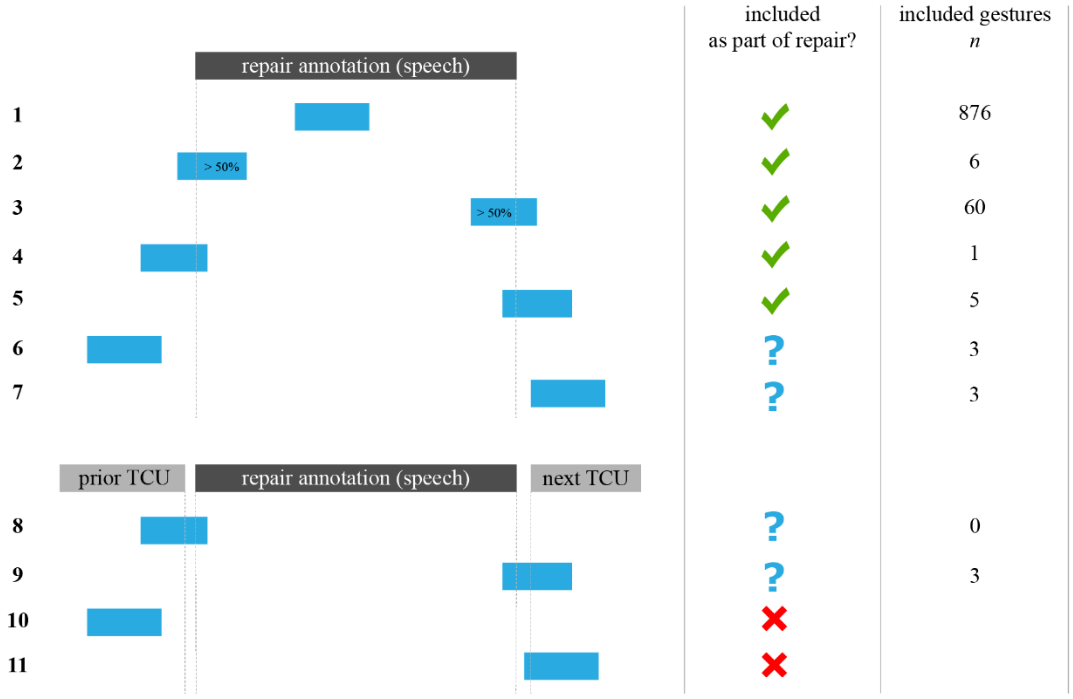

Figure S1.4. Schematic visualization of gestures (not) considered to be part of repair annotations

S1.5 Operationalization of gesture effort: more examples

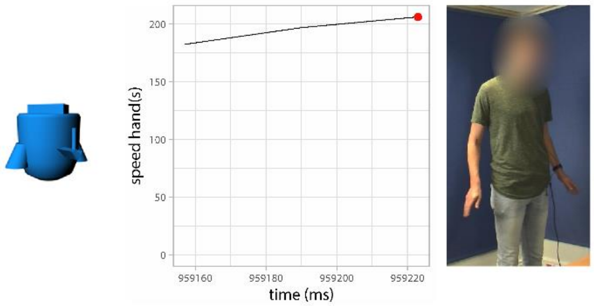

Figure S1.5a. An example of a gesture which is produced by a director as part of a repair solution in response to a restricted offer. The accompanying speech is 'ja ja ja' [*yes yes yes*], thereby confirming the restricted offer which contained a similar gesture. The hands model the subparts on the left (fully visible) and right side (hardly visible) of the Fribble by keeping them still next to either side of his body (number of submovements: 1).

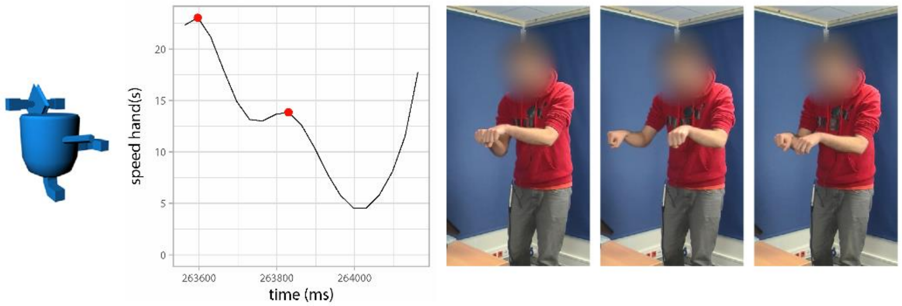

Figure S1.5b. An example of a gesture which is produced by a matcher as part of a repair initiation of the type restricted offer, in response to an unclear turn of the director who described the subparts at the top of the Fribble. The gesture depicts the blocks on the left and right side of the tilted square; the hands start together in the middle, then move sideward and then back to the middle again (number of submovements: 2). The accompanying speech was: "(..) is het een een rechthoek wat in het midden staat waarbij dus uh dat die uh twee objecten met elkaar verbindt?" [*(..) is it a a square that stands in the middle where so uh that connects those uh two objects with each other*?].

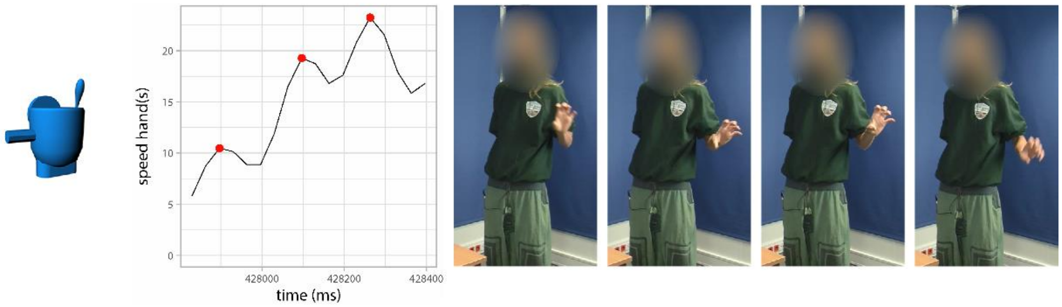

Figure S1.5c. An example of a gesture which is produced by a matcher as part of a repair initiation of the type restricted offer, while saying: "ja redelijk uh blokkerige staaf?" [*yes quite uh blocky bar?*]. The left-handed gesture depicts the subpart on the left side of the Fribble. The "blocky" aspect is depicted by thrusting the hand sideward in a couple of back-and-forth movements (number of submovements: 3).

S2 Results details

In the main text we have reported the analyses for the multimodal division of effort. Here we elaborate on the potential of analyzing the division of effort for the spoken and gestural modality separately. We postulate that, given that people had multiple modalities available to them in which they could express themselves – and the fact that we know that people indeed frequently did so in both modalities – it makes little sense to analyze efforts from a unimodal perspective. For the gestural modality this is further complicated due to the nature of the data. Whereas all repair turns consist of speech (with a minimum of 2 orthographic characters), this is not the case for gestures (across all repair types and sequential positions, the median number of submovements is 0). Thus, we have a smaller set of repair sequences for which we could assess the division of gestural effort at all, and many cases with a highly skewed division of gestural effort (0% versus 100%).

Yet some readers might wonder about the division of verbal effort, and whether those patterns are in line with the earlier findings by Dingemanse et al.[18]. Indeed, we too find that the proportional verbal cost paid by the person initiating repair varies as a function of the repair type. The proportional speech effort in the repair initiation was higher for restricted requests compared to open requests ($\beta = 0.17$, $SE = 0.06$, $t = 3.08$, $p = .002$), and higher for restricted offers compared to restricted requests ($\beta = 0.37$, $SE = 0.04$, $t = 9.96$, $p < .001$), as revealed by mixed effects models (with random intercepts for dyads). Thus, this replication shows that the *division of* labor principle appears to be robust in terms of speech efforts, but it is only now that we have checked the *multimodal* division of labor for *multimodal* data that we can take Dingemanse et al.'s[18] findings to hold water for interactions in their true multimodal form.

References

1. Schegloff, E. A. When 'others' initiate repair. *Appl Linguist* **21**, 205–243 (2000).
2. Schegloff, E. A., Jefferson, G. & Sacks, H. The preference for self-correction in the organization of repair in conversation. *Language* **53**, 361–382 (1977).
3. Dingemanse, M., Kendrick, K. H. & Enfield, N. J. A Coding Scheme for Other-initiated Repair Across Languages. *Open Linguistics* **2**, (2016).
4. Clayman, S. E. Turn-Constructional Units and the Transition-Relevance Place. in *The Handbook of Conversation Analysis* 151–166 (John Wiley & Sons, Ltd, 2013). doi:10.1002/9781118325001.ch8.
5. Couper-Kuhlen, E. & Selting, M. *Interactional Linguistics: Studying Language in Social Interaction.* (Cambridge University Press, 2017).
6. Schegloff, E. A. *Sequence Organization in Interaction: A Primer in Conversation Analysis*. vol. 1 (Cambridge University Press, 2007).
7. Koo, T. K. & Li, M. Y. A Guideline of Selecting and Reporting Intraclass Correlation Coefficients for Reliability Research. *Journal of Chiropractic Medicine* **15**, 155–163 (2016).
8. Lücking, A., Bergman, K., Hahn, F., Kopp, S. & Rieser, H. Data-based analysis of speech and gesture: the Bielefeld Speech and Gesture Alignment corpus (SaGA) and its applications. *Journal on Multimodal User Interfaces* **7**, 5–18 (2013).
9. Lücking, A., Ptock, S. & Bergmann, K. Assessing Agreement on Segmentations by Means of Staccato, the Segmentation Agreement Calculator according to Thomann. in *Gesture and Sign Language in Human-Computer Interaction and Embodied Communication* (eds. Efthimiou, E., Kouroupetroglou, G. & Fotinea, S.-E.) 129–138 (Springer Berlin Heidelberg, 2012).
10. Feng, G. C. Intercoder reliability indices: disuse, misuse, and abuse. *Quality & Quantity* **48**, 1803–1815 (2014).
11. Feinstein, A. R. & Cicchetti, D. V. High agreement but low Kappa: I. the problems of two paradoxes. *Journal of Clinical Epidemiology* **43**, 543–549 (1990).
12. Dingemanse, M., Blythe, J. & Dirksmeyer, T. Formats for other-initiation of repair across languages: An exercise in pragmatic typology. *Studies in Language* **38**, 5–43 (2014).
13. Bekke, M. ter, Drijvers, L. & Holler, J. The predictive potential of hand gestures during conversation: An investigation of the timing of gestures in relation to speech. (2020) doi:10.31234/osf.io/b5zq7.
14. McNeill, D. *Hand and Mind: What Gestures Reveal about Thought.* (University of Chicago Press, 1992).
15. Wagner, P., Malisz, Z. & Kopp, S. Gesture and speech in interaction: An overview. *Speech Communication* **57**, 209–232 (2014).
16. Healey, P. G. T., Plant, N. J., Howes, C. & Lavelle, M. When words fail: Collaborative gestures during clarification dialogues. in *2015 AAAI Spring Symposium Series* 23–29 (2015).
17. Holler, J. & Wilkin, K. Co-Speech Gesture Mimicry in the Process of Collaborative Referring During Face-to-Face Dialogue. *J Nonverbal Behav* **35**, 133–153 (2011).
18. Dingemanse, M. *et al.* Universal Principles in the Repair of Communication Problems. *PLOS ONE* **10**, e0136100 (2015).
